# Complete genome sequence and pathogenesis of bovine viral diarrhea virus JL-1 isolate from cattle in China

**DOI:** 10.1186/1743-422X-11-67

**Published:** 2014-04-08

**Authors:** Shuqin Zhang, Bin Tan, Yulin Ding, Fengxue Wang, Li Guo, Yongjun Wen, Shipeng Cheng, Hua Wu

**Affiliations:** 1 State key Laboratory for Molecular Biology of Special Economic Animals-Institute of Special Animal and Plant Sciences, Chinese Academy of Agricultural Sciences, Changchun 130112, China; 2 College of Veterinary Medicine, Inner Mongolia Agricultural University, Hohhot 010018, China; 3 Sinovet (Beijing) Biotechnology Co.,Ltd., Beijing 100085, China

**Keywords:** Bovine viral diarrhea virus (BVDV), Complete genome, Pathogenesis, BVDV-1b

## Abstract

**Background:**

Bovine viral diarrhea virus (BVDV) is a pathogen found worldwide in calves. It can cause significant economic losses in agriculture. Many BVDV strains have been isolated in China. However, the pathogenesis and complete gene characteristics of BVDV isolate have yet not been reported in China. Here, a BVDV isolate was isolated and its pathogenesis and complete genome were studied.

**Results:**

A new isolate of bovine viral diarrhea virus, named JL-1, was isolated from the spleen of a sick cow with diarrhea using MDBK cell culture. The complete genome of JL-1 is 12,276 nucleotides and contains a 5′-UTR of 382 nucleotides, a 3′-UTR of 188 nucleotides, and a large ORF encoding a polyprotein consisting of 3,901 amino acids. Genomic comparison and phylogenetic analyses of complete genomic sequence clearly showed that JL-1 fell into the BVDV-1b subtype. The result of pathogenesis of JL-1 strain showed that all infected calves developed clinical signs of elevated rectal temperatures, decreased leucopenia, and viral discharge. Viral antigen was detected in infected animal tissues using immunohistochemistry. Animals in the mock were normal. These results demonstrated that BVDV JL-1 was a virulent strain.

**Conclusions:**

This is the first study to report the pathogenesis and complete gene characterization of the BVDV strain in China. This report may set a good foundation for further study of BVDV in China.

## Background

Bovine viral diarrhea virus (BVDV), the etiological agent of bovine viral diarrhea/mucosal disease, is widespread and one of the most economically important diseases of cattle. BVDV-associated diseases can range from clinically undetectable to severe. They may involve the respiratory, enteric, reproductive, immune, and endocrinee systems [1,2]. BVDV is a small, enveloped virus with a single-stranded, positive-sense RNA genome, that is ~12.5kb in length and contains a 5′- untranslated region (5′-UTR), a single open reading frame and a 3′-untranslated region (3′-UTR) [3]. According to a comparison of the 5′-UTR and the Npro- and E2-encoding sequences, BVDV isolates are divided into two genotypes (BVDV1 and BVDV2) with several subtypes in each group [4,5]. The 5′-UTR genomic region provides meaningful inferences as this region has the highest degree of sequence conservation and is effectively amplified by RT-PCR [6,7]. BVDV viruses of both genotypes are divided into cytopathic (cp) and non-cytopathic (ncp) biotypes, based on the ability of the virus to cause cytopathogenicity in tissuecum culture. Noncytopathic BVDV is the only biotype that has been observed clinically or experimentally to cause BVDV persistent infection. In addition, BVDV are lymphotrophic and the immunosuppression that follows infection results in increased severity in secondary infections. The interaction of BVDV with secondary pathogens is thought to be one of the contributing factors in bovine respiratory disease complex (BRD) [8-10]. BVDV associated disease can range from clinically inapparent to severe and can involve the respiratory, enteric, reproductive, immune and endocrine systems.

Infection with BVDV poses a major threat to the cattle industry. This threat can be minimized through the use of commercially available BVDV vaccines. BVDV vaccines have played a significant role in the prevention of various clinical disease syndromes associated with BVDV infections, as well control of the spread of the virus [11,12]. Now, many countries using vaccination strategies to limit losses due to BVDV. The BVDV 1a and BVDV2a were the main subtypes in modified live virus (MLV) and killed viral vaccines. Only one killed vaccine contains BVDV1b subtype (NY-1 strain) [13]. To evaluate potency of the vaccines, it is unavoidable to need an appropriate virulent virus strain. BVDV was first isolated from calves in China 1980 [14,15]. To date, many BVDV strains were isolated in china, the BVDV circulating in Chinese calves is mainly BVDV1b [16,17]. However, there are no commercial BVDV vaccines in the market of China at present, and the pathogenic BVDV isolate was not reported previously in China. At the basic biology level, the study of virulence variation is important to understanding the mechanisms behind pathology [18]. The purpose of this work was isolate and examine the virulence of BVDV JL-1 strain, which would contributes to the research of this disease and development of BVDV vaccine candidate.

## Results

### Isolation and identification

**Figure 1:**
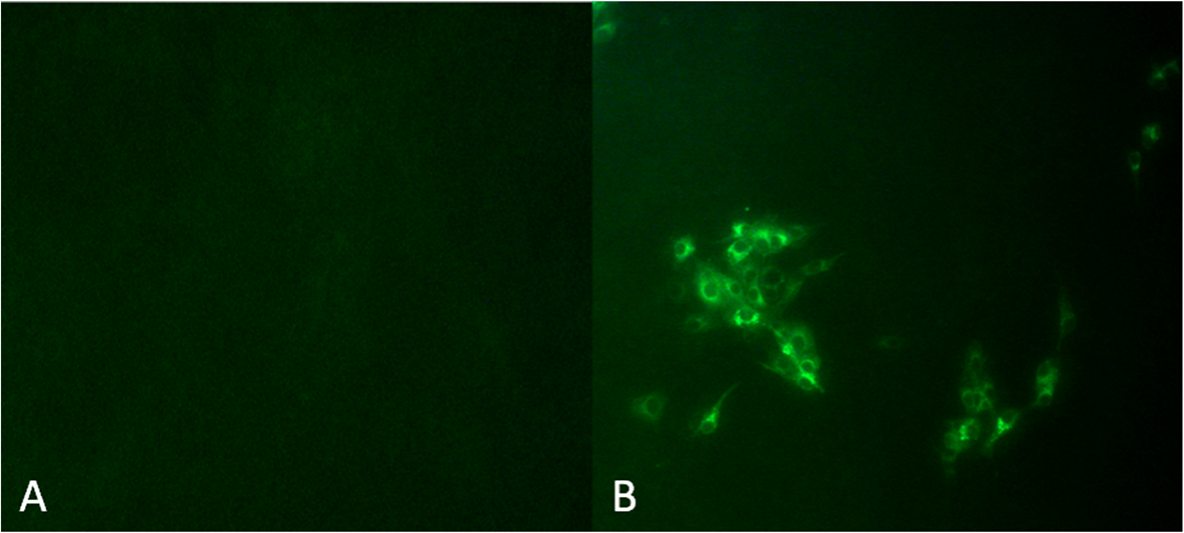
**IFA identification of virus isolate cultured in MDBK cells. A**. Non-infected MDBK cells; **B**. BVDV JL-1 Isolate-infected MDBK cell (200×).

Homogenates of tissues samples were cultured and passaged in MDBK cells, and no cytopathic effects (CPE) was observed in MDBK cell after 4 passages. The presence of BVDV isolates in MDBK cells has been confirmed by FITC-labeled BVDV antibody. The specific green signals of FITC were detected in the infected group, and the mock group had no fluorescence signals (Figure 1), then one viral isolate was obtained and named strain BVDV JL-1.

### Sequence and phylogenetic analysis of the complete genome

**Figure 2:**
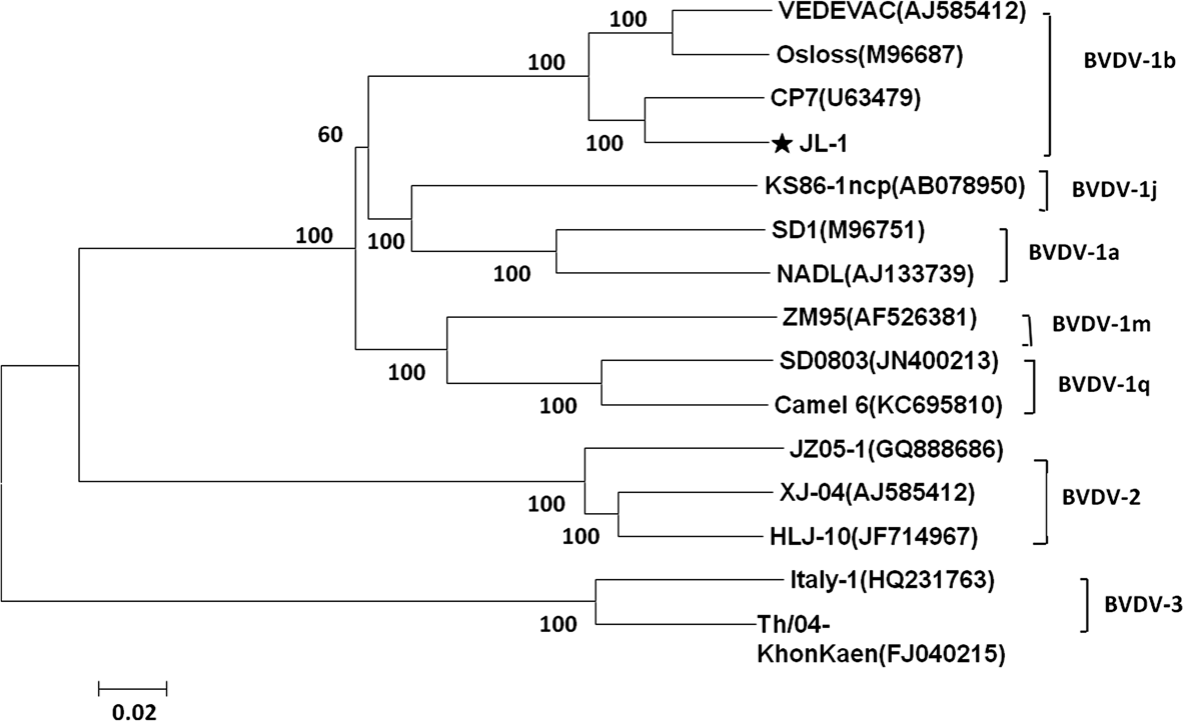
**Phylogenetic tree analysis of the E2 was created using the nucleotide sequences of the JL-1 isolate.** The phylogenetic tree was prepared using the Neighbor-Joining method and bootstrap testing. Numbers over branches indicate the percentage of 1,000 bootstrap replicates that support each phylogenetic branch.

The complete genome of JL-1 was 12,276 nucleotides in length. It contains 5′-UTR of 382 nucleotides, a 3′-UTR of 188 nucleotides, and a large ORF encoding a polyprotein consisting of 3,901 amino acids. The complete genomic sequence of JL-1 has been submitted to GenBank under accession number KF501393. The nucleotide and amino acid sequences of the structural and nonstructural proteins of JL-1 were most similar to those of strain CP7 (a prototype of BVDV-1b). The nucleotide identity of the complete genome was 67.6–93.2% between JL-1 isolate and BVDV1, BVDV2, and BVDV3 strains. A 93.2% identity of JL-1 isolate and CP7 genome was observed. The similarity between JL-1 isolate and other BVDV strains with respect to deduced amino acids ranged from 72.4–95.3%. A phylogenetic tree was constructed by comparing the E2 sequence of JL-1 and other pestiviruses obtained from GenBank. These results of the analysis showed that the JL-1 strain was classified as BVDV1b with 100% bootstrap value (Figure 2).

### Pathogenesis of BVDV JL-1 strain

All 5 calves inoculated with BVDV JL-1 developed moderate clinical signs associated with BVDV infection, including depression, fever, leucopenia, and viremia. The control calves were normal.

**Figure 3:**
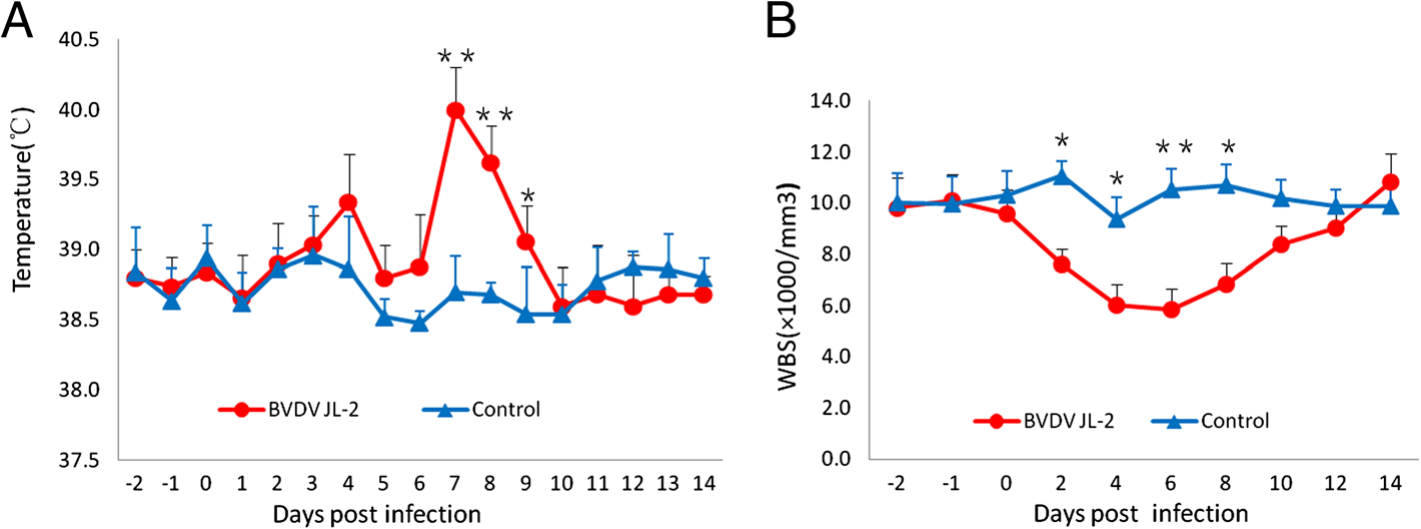
**The clinical observation following inoculated with BVDV JL-1 virus. A**. elevated rectal temperatures; and decrease of white blood cell counts **B**. The error bars represent standard deviation. Statistical values are *p<0.05 and **p<0.01.

The rectal temperatures of calves were measured daily from day −1 (before inoculation) to day 14 (after inoculation). All treated calves (5/5) showed elevated rectal temperatures, with peak temperatures appearing on days 7–8 after inoculation. Two calves had rectal temperatures higher than 40.0°C on several days. The control calves did not develop fevers at any point in time after treatment (Figure 3A).

White blood cell counts of treated calves started decreasing from day 2 after inoculation. The mean WBC counts for the control group dropped significantly from 9.8×1000 per mm^3^ on day 0 to 5.9×1000 per mm^3^ on days 4 and 6 after inoculation, a decrease of approximately 40% (Figure 3B). The mean WBC counts in the control group did not decrease after inoculation, and they were significantly higher (*P*<0.05) than that of the control group on days 2–8 post-inoculation.

Nasal swab samples were collected from all calves from day −1 to day 14 after inoculation and viral shedding was assessed. All treated calves shed BVDV on multiple days from day 4 to day 10 after inoculation. Buffy-coat samples were collected from the blood taken every other day to day 14 after inoculation. The isolation of the virus from the blood was conducted to assess viremia in the calves. Results showed that all treated calves had viremia and BVDV was isolated from blood from different cows on days 4–10. Sera obtained before inoculation with BVDV JL-1 and on day 14 was subjected to neutralization testing. Results showed that all of the virus-infected calves developed virus-neutralizing antibodies by day 14 (Table 1).

**Table 1:** Detection of virus isolation and Serum neutralization antibody titres from calves experimentally inoculated with BVDV JL-1 strain

**Animal no.**	**No. of days that samples virus was isolated/total no.**			**SN titres to BVDV**
	**Virus isolation (buffer coat)**	**Virus isolation (Nasal swab)**	**RT-PCR (Nasal swab)**	
Calve 170144	2/8	3/15	4/15	16
Calve 170115	2/8	4/15	6/15	32
Calve 170166	3/8	5/15	7/15	64
Calve 183117	3/8	3/15	5/15	48
Calve 183155	1/8	3/15	5/15	8

**Figure 4:**
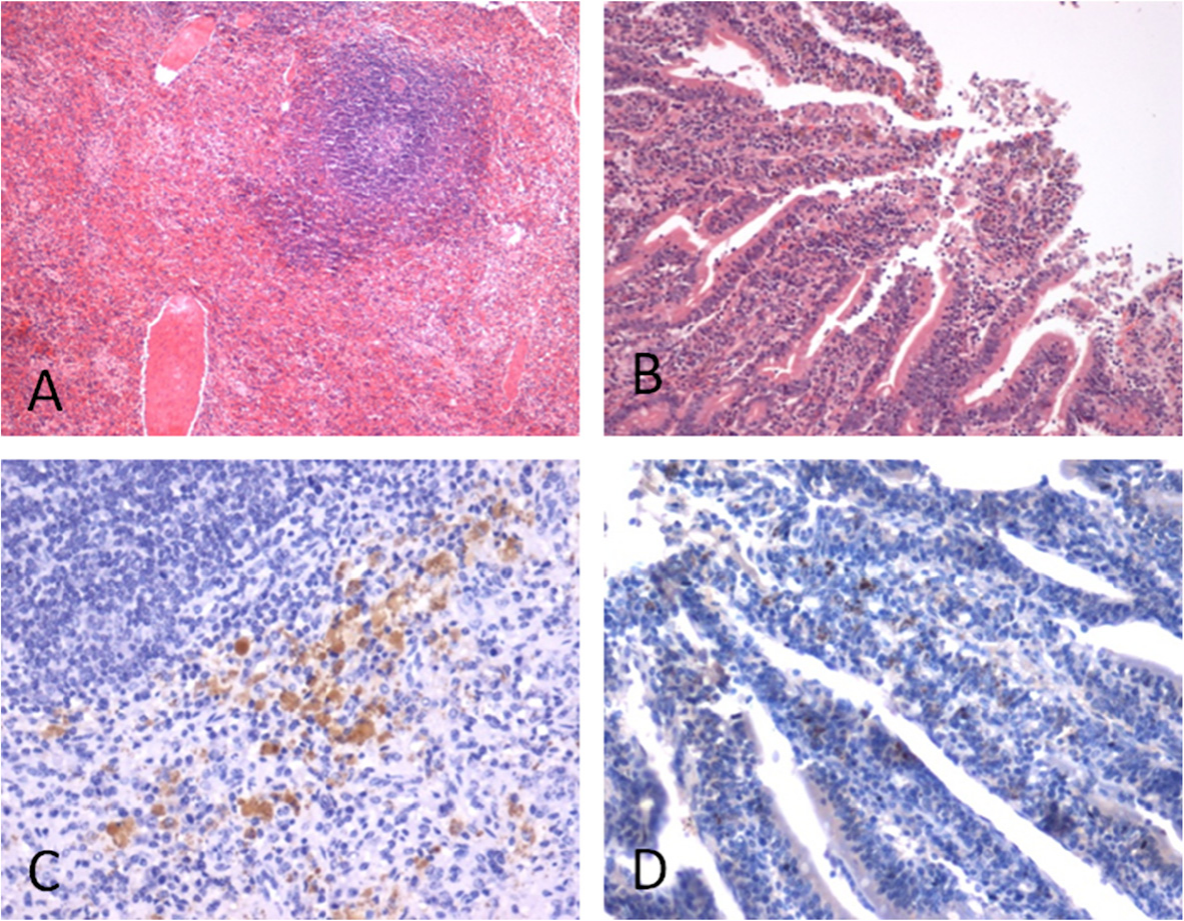
**The pathology detection following inoculated with BVDV JL-1.** Histopathology appearances of H&E-stain **(A and B)**, **A**. spleen, showing the significantly wided periarterial lymphatic sheath and lymphocytes depletion in red pulp (100×); **B**. intestines: showing hemorrhage and shedding in the small intestine villi (100×). Immunohistochemistry (IHC) detection of BVDV antigen **(C and D)**. **C**. spleen, virus antigens in mononuclear cells randomly scattered in splenic red pulp (200×); **D**. virus antigens in epithelium of intestines (200×).

The primary morphological changes were observed in the digestive tract and lymphoid tissue. Briefly, there was a little reduction the thickness of the intestine and mild hyperemia, and edema of the small intestinal mucosa and swelling and hemorrhage of mesenteric lymph nodes and the gut-associated lymphoid tissue were observed. The small intestine villi showed mild hemorrhage and shedding (Figure 4A). Lymphoid depletion was the most prominent within the germinal centers of follicles of the lymphoid node and in the periarteriolar lymphoid sheath (Figure 4B). In IHC staining, the distribution of BVDV-specific antigen was consistently found in the epithelia of the intestines and in mononuclear cells randomly scattered in splenic red pulp (Figure 4C and D). No pathological damage was found in the control calves.

## Discussion

In this study, a new BVDV JL-1 strain was isolated from calves afflicted with BVD, a recently discovered BVDV present in northeastern China. The identification of the isolate has been confirmed by IFA. Many other studies have demonstrated that the BVDV-1b subgenotype is the most common of the BVDV-1 field isolates. The trend of relative increase in BVDV-1b isolates was found not only during comparison of different geographic regions but also during comparison of the isolates found over the past 20years in one diagnostic laboratory [19,20]. In this study, the phylogenetic relationships among BVDV JL-1 and other BVDV wild-type strains from GenBank were clarified. Genetic and phylogenetic analysis showed that the virus BVDV JL-1 belonged to BVDV1b, and a high degree of identity was detected among all studied wild-type strains in China.

The present pathogenesis study was performed upon conventionally reared calves. Results indicated that BVDV JL-1 can induce mild clinical disease. Clinical presentation after infection with BVDV JL-1 was similar to that observed after infection with the BVDV NY-1 strain. Clinical manifestations included mild short-term pyrexia (basal temperatures between 39.2 and 40.0°C for 1–3days), pronounced and prolonged febrile response, pronounced reduction in circulating white blood cells (>40%), and viral excretion in the buffy coat. All of the virus-infected calves developed virus-neutralizing antibodies by day 14. Strain NY-1 belongs to the BVDV1 pestivirus species (subgenotype BVDV1b). It is the first noncytopathic BVDV strain to be isolated. In recent years, the NY-1 strain has become an accepted laboratory reference strain. It has also been offered as a challenge strain for vaccine efficacy studies [18].

The clinical symptoms observed in calves from which BVDV JL-1 was isolated mainly showed anorexia, diarrhea, dehydration, and marasmus. The present pathogenesis study, which was performed upon BVDV JL-1, only mild clinical signs associated with BVDV infection were observed. These included depression, fever, leucopenia, and viremia. None of the treatment calves showed diarrhea. This might be related to the immune state and age of experimental animals. In this report, the BVDV JL-1 virus isolates were confirmed to be free of contamination by viruses, mycoplasmas, bacteria, and fungi. The experimental animals were 6 to 9 months old and immunocompetent. No secondary infections occurred during the animal experiment. Histopathological observation showed lymphoid depletion and shedding of the villi of the small intestine. If the treatment calves experienced a secondary infection at that time, the results led to an exacerbation of the inflammatory response and to the development of more intense clinical symptoms and lesions than those observed in healthy animals [21]. Various authors have demonstrated that BVDV can induce lymphopenia and has a range of effects on the immune response. These can allow secondary infection to take place [22,23].

Ever since the first isolation of BVDV in 1980, no effective measures have been developed to control the spread of BVDV in China. This is the first report of a pathogenic BVDV strain in China, here called BVDV JL-1. It is an NY-1 strain, so infection of immunocompetent adult calves can cause clinical manifestations. These findings may contribute to the development of a vaccine for the prevention and control of BVDV in China.

## Conclusions

In this study, a BVDV JL-1 strain was successfully isolated from cattle in china. Genomic comparison and phylogenetic analyzes showed that JL-1 fall into BVDV-1b subtype, the predominant genotype in China. Pathogenic assay show its remains virulent and can be used as a BVDV challenge virus to evaluate the efficacy of BVDV vaccines. This report will be a good beginning for further studies on BVDV in china.

## Methods

### Virus isolation

Field samples came from calves at field of Jilin province (China), which clinical symptoms showed anorexia, diarrhea, dehydration and marasmus. The tissue samples were collected from the necropsies of one calve and then stored at −80°C. The virus isolation was conducted as described previously [24,25]. Briefly, Homogenates containing 1g of each tissue (including spleen, lung, thymus, intestine and Lymph node) samples and 10 mL of Dulbecco’s modified Eagle’s medium with antibiotics were sonicated and centrifuged. A monolayer of MDBK cell was infected with the homogenates in a 24-well culture plate. The cell cultures were frozen and thawed three times and passaged two to three times at five days interval. The every passage of MDBK cultures were observed for 5days with presence or absences of CPE recorded.

### RT-PCR detection and complete genomic sequence analysis

Total RNA was extracted from field tissue samples and cell culture fluids of virus isolates using TRIZOL (Invitrogen, China) according to the manufacturer’s instructions. The extraction was accomplished using 250 μl of samples and 750 μl of TRIZOL. The RNA was resuspended in 30 μl DEPC-treated water. The extracted RNA was reverse-transcribed using the M-MLV Reverse Transcriptase Kit (Invitrogen, USA) as specified by the manufacturer. Twelve primer sets were designed to amplify overlapping regions of the complete BVDV genome. The amplified fragments were harvested and cloned into pMD–18T vector (TaKaRa, China). Three recombinant clones were submitted to DNA sequencing (Invitrogen Biotech, Beijing, China). The retrieved sequences were edited and trimmed with the EditSeq program in the DNASTAR software. Clustal W was used to align the nucleotide sequences. Phylogenetic analysis was done by the distance-based clustal W method using software MEGA 4.1.

### Immunofluorescence assay (IFA)

A 96 well microtitre plate (Costar, NY, USA) was seeded with MDBK cell in DMEM with 8% ES, and cultivated at 37°C in 5% CO_2_ overnight until 70-80% confluence. The cultures were then inoculated with 20-fold diluted field BVDV isolates at 3 passage. The uninfected cultures in left rows of the plate were taken as the negative controls. After 72hrs incubation in 37°C, 5% CO_2_ atmosphere, the plate was fixed in 80% cold acetone/methanol, and then washed with PBS, FITC-conjugated Anti-Bovine Viral Diarrhea Virus (BVDV) polyclonal antiserum. (VMRD, USA) was added to the plate, and then followed by 30 min incubation in a 37°C humid box. After 3 times washes with PBS, a 50% glycerol in PBS was added to each well. Two infected wells were treated as a positive control to confirm viral growth. Fluorescence signal was observed using an fluorescence inverted microscope (Zeiss Axioskop-40, Germany).

### Infection of animals

Ten healthy calves (6 to 9-month-old) were obtained from a calve farm that was negative for BVDV infections. All animals were confirmed to be free of BVDV,IBRV,BPI3, Mycoplasma bovis infections using enzyme-linked immunosorbent assay (ELISA) kits for the detection of antibodies against BVDV, IBRV, BPI3, or M. bovis (Idexx Labs Inc., American) and by reverse transcription-PCR (RT-PCR) or PCR for viral nucleic acid detection [26-28]. All animal work and experimental procedures were conducted with an approval of Institutional Animal Care and Use Committee of Jinlin University, China. The 10 calves were divided randomly into treatment group and control group, with 5 animals in each group. Each calve of the treatment group was inoculated intranasally (IN) with ~6×10^7.0^ tissue culture infective doses (TCID_50_) of BVDV JL-1 virus. The animals in control group were inoculated IN with DMEM. All animals were monitored daily for clinical signs as described previously [29]. Clinical assessments were made at the same time each morning by investigators who were blinded to the treatment groups. Clinical signs included depression, nasal discharge, diarrhea, coughing and rectal temperatures. EDTA-blood samples from calves were collected at days−2 to 0 prior to inoculation and days 2, 4, 6, 8, 10, 12, 14 post-inoculation (dpi) and used to count white blood cells. Additionally, at day 0, 2, 4, 6, 8, 10, 12, 14 post-inoculation (dpi), heparin blood was taken for buffy-coat preparations to test for viremia in the challenged calves. Deep nasal swab specimens were obtained from one day prior to challenge through 14 days post inoculation. The procedure to isolate BVDV from buffy-coat samples was conducted as described previously [30]. Sera obtained before inoculation with BVDV JL-1 and on day 14, Serum sample were tested for neutralizing antibodies to BVDV JL-1 using standard microtitration procedures [31].Two calves out from each group were necropsied at 14 DPI, and their tissue samples were collected and fixed in 10% neutral buffered formalin and processed for histopathological examination following hematoxylin and eosin (H&E) staining and immunohistochemistry.

The two groups (treatment and control) were analyzed and compared with respect to the primary clinical signs including rectal temperature, nasal and ocular discharges,diarrhea, leukopenia and virus shedding, using GraphPad Prism (version 4.0) software. The level of statistically significant difference was set at p<0.05.

## Competing interests

The authors declare that they have no competing interest.

## Authors’ contributions

SQZ, BT, YLD, LG, YJW carried out the immunoassays. “SQZ, FXW carried out the molecular genetic studies. SQZ, SPC, HW conceived of the study, and participated in its design and coordination and helped to draft the manuscript. All authors read and approved the final manuscript.”
